# Association of Physician Group Participation in Accountable Care Organizations With Patient Social and Clinical Characteristics

**DOI:** 10.1001/jamanetworkopen.2018.7220

**Published:** 2019-01-18

**Authors:** Rachel M. Werner, Genevieve P. Kanter, Daniel Polsky

**Affiliations:** 1 Center for Health Equity Research and Promotion, Crescenz VA Medical Center, Philadelphia, Pennsylvania; 2 Division of General Internal Medicine, Perelman School of Medicine, University of Pennsylvania, Philadelphia; 3 Leonard Davis Institute of Health Economics, University of Pennsylvania, Philadelphia

## Abstract

**Importance:**

Accountable care organizations (ACOs) may increase health care disparities by excluding physician groups that care for socially and clinically vulnerable patients.

**Objective:**

To estimate the association between the patient characteristics of a physician group and the group’s participation in a newly formed ACO.

**Design, Setting, and Participants:**

This retrospective cohort study investigated a 20% random sample of US Medicare fee-for-service beneficiaries attributed to physician groups identified in Medicare claims before ACO participation from January 1, 2010, through December 31, 2011. Physician groups that participated and did not participate in the Medicare Shared Savings Program (MSSP) from January 1, 2012, through December 31, 2014, were identified in the Medicare MSSP 2014 provider file. Data analyses were conducted from September 1, 2017, to March 30, 2018.

**Exposures:**

Using multivariable regression, the association between physician group participation in the MSSP and the group’s patients’ characteristics before ACO formation was estimated focusing on measures of the vulnerability of the group’s patients. All ACO-participating physician groups were compared with ACO-nonparticipating physician groups for reference, and estimates were made at the physician and patient level.

**Main Outcomes and Measures:**

Percentage of a physician group’s patient panel that was socially vulnerable (based on race, dual Medicare and Medicaid enrollment, or living in high-poverty zip code) or clinically high risk.

**Results:**

Among 67 891 physician groups caring for 5 394 181 patients, 7215 physician groups (10.6%) participated in an MSSP ACO by 2014. Comparing mean percentages across practices, the patients of non–ACO-participating physician groups, more patients of ACO-participating physician groups were black (mean percentage across practices, 12.1% vs 10.6%), dually enrolled in Medicare and Medicaid (23.0% vs 19.3%), living in poverty (10.7% vs 11.1%), and high risk (34.2% vs 30.2%). After adjustment, physician groups that participated in an ACO had 5.1 percentage points (95% CI, 0.1-10.0 percentage points; *P* = .05) more dually enrolled patients and 4.0 percentage points (95% CI, 1.9-6.1 percentage points; *P* < .001) more high-risk patients. At the patient level, patients who were at high risk were more likely to be attributed to a group that became part of an ACO, with 4.5 percentage points (95% CI, 0.5-8.5 percentage points; *P* = .03) more high-risk patients being attributed to an ACO, but other associations were not statistically different from zero.

**Conclusions and Relevance:**

Accountable care organizations may be an effective approach to target care among high-risk patients. In this study, physician groups that participated in the MSSP ACO program cared for more clinically vulnerable patients than did nonparticipating groups, and ACO-participating physician groups cared for an equally large number of socially vulnerable patients compared with nonparticipating physician groups.

## Key Points

**Question:**

Are physician groups that care for socially and clinically vulnerable patients less likely to participate in accountable care organizations?

**Findings:**

This cohort study of US physician groups from 2010 through 2014 found that groups participating in Medicare Shared Savings Program accountable care organizations cared for more clinically high-risk patients than did nonparticipating physician groups. There was no difference in patient social vulnerability between the 2 groups.

**Meaning:**

The findings suggest that Medicare’s Shared Savings Program accountable care organizations may be an effective approach to target care coordination among high-risk patients.

## Teaser

This cohort study evaluates Medicare claims data and Medicare Shared Savings Program files from 2010 through 2014 to investigate the association between US physician groups’ participation in an accountable care organization and the clinical and social characteristics of their patient populations.

## Introduction

Accountable care organizations (ACOs) are networks of health care practitioners that take on responsibility for managing the health care of a group of patients across the full continuum of health care settings and are held financially accountable for providing high-quality and low-cost care. Accountable care organizations are an important and far-reaching recent change to health care with exponential growth over the past few years, covering more than 32 million lives by 2016.^1^

Financial accountability is based on the sharing of savings, which is determined by whether the actual expenditures of the participating health care practitioners’ assigned patient population are below a benchmark established by the prior expenditures of their assigned patients. Early evidence suggests that this ACO incentive structure can improve quality and constrain costs.^2,3,4,5,6,7,8,9,10^

This incentive structure may also drive providers’ choice to enter into an ACO arrangement. Physician groups with patient panels that would most likely benefit from the coordination and management that the ACO model rewards may have the greatest incentives to enter into ACO contracts. These groups might include practices caring for a large number of patients with clinically complex conditions. If practices with more patients with complex conditions were more likely to participate in an ACO, documented improvements under the ACO model might appropriately be directed toward patients who would benefit the most.

However, in reality, the choice to be in an ACO may depend on the providers’ capabilities to incorporate management tools to help manage and coordinate care for patients with complex needs. These tools often require capital that might not be available to practices that serve poorly resourced populations, populations that often both have complex clinical needs and are socially vulnerable. On one hand, socially vulnerable groups (such as racial/ethnic minorities and patients living in impoverished neighborhoods) have much to gain from ACOs because they have a higher prevalence of many chronic health conditions, receive poorer quality of care, and experience worse health outcomes.^11,12^ On the other hand, because care for vulnerable patients has historically been concentrated among relatively few providers who tend to have fewer financial and health care resources and perform worse on most traditional quality metrics,^13,14,15,16^ ACOs may thus have little incentive to include providers that do not perform well on quality metrics and would require additional capital and resources to allow for the efficient management of population health. These realities raise concerns that ACOs could widen existing health care disparities.^17,18,19^

Previous research has shown that ACOs have predominantly formed in geographic areas with a higher proportion of well-insured patients.^20^ Additional research found that even after accounting for these geographic differences in where ACOs form, ACOs are less likely to include physicians working in areas that are more densely populated by low-income, low-educated, racial minority patients.^21^ Thus, providers serving a disproportionate share of vulnerable patients may be systematically excluded from ACOs despite the fact that these patients may stand to gain the most from a well-functioning ACO model.

However, the research to date has not examined whether the characteristics of physicians’ patients are associated with which physician groups participate in ACOs. Thus, we estimated the association between physicians’ patient characteristics and their likelihood of participating in a newly formed ACO. We focused on one of the best known and largest experiments with ACOs, the Medicare Shared Savings Program (MSSP),^22^ which contracts with 561 ACOs and covers 10.5 million lives.^23^

## Methods

Our overall approach was to estimate the likelihood of a physician group participating in an ACO in 2014 based on the characteristics of that physician group’s patients before ACO formation, focusing on measures of the vulnerability of the group’s patients. All ACO-participating physician groups were compared with ACO-nonparticipating physician groups for reference. The institutional review board of the University of Pennsylvania approved this study and waived informed consent.

### Data

We identified physicians participating in MSSP ACOs from January 1, 2012 (the first year of MSSP), through December 31, 2014, using Medicare’s MSSP provider file. To identify patients served by these physicians and ACO-nonparticipating physicians at baseline (prior to MSSP), we used 2010 and 2011 Medicare physician claims for an approximately 20% random sample of Medicare fee-for-service beneficiaries. These claims data were combined with the Medicare Beneficiary Summary File to determine Medicare enrollment in Part A, Part B, and Medicare Advantage. These Medicare data were supplemented with American Community Survey data for beneficiary zip code–level poverty and the SK&A Office Based Physician file for physician group characteristics. Data analyses were conducted from September 1, 2017, to March 30, 2017.

### Study Cohort

We started with all Medicare fee-for-service beneficiaries enrolled in Part A and Part B, and following MSSP methodology,^24^ we attributed beneficiaries to a physician taxpayer identification number, which approximates physician groups. We attributed beneficiaries with at least 1 primary care service from a physician. Each beneficiary was attributed to the taxpayer identification number where the beneficiary received the most primary care services (measured by Medicare-allowed charges) furnished by primary care physicians as defined by the MSSP. Attribution was done annually during the pre-ACO period (2010 and 2011) and was done for all physician groups regardless of whether they later participated in the MSSP. We then excluded group-year observations with 10 or fewer attributed beneficiaries in a year to ensure that each physician group had a sufficiently large panel of patients from whom to calculate panel characteristics. This step excluded 3.6% of beneficiaries in 46.2% of physician groups. We then averaged panel characteristics from 2010 and 2011 to produce our final study cohort, which was a physician group–level cohort with each group represented once.

### Variables

Our outcome of interest was an indicator of whether each physician group participated in an ACO in the MSSP by 2014. Our main independent variables were 4 physician group–level measures describing each group’s patient panel before the initiation of ACOs (2010-2011). These 4 variables were the percentage of a group’s patient panel that was black; the percentage of a group’s patient panel that was dually enrolled in Medicare and Medicaid; the average zip code–level poverty rate for patients in a group; and the percentage of a group’s patient panel with a Charlson Comorbidity Index of 3 or greater. The Charlson Comorbidity Index measures the burden of disease and the prognostic associations with that disease burden. It considers which of the 17 diseases of interest a person has, assigns a point score to each based on mortality risk, and sums them to generate a score of disease burden. The final risk scores range between 0 and 41, with higher values associated with greater mortality risk over 10 years.^25^ We included the following physician group–level variables from the SK&A Office Based Physician file as covariates: the number of physicians in the physician group, the percentage of physicians who were specialists, the percentage affiliated with a health system, and the percentage who were hospital based.

### Statistical Analysis

Our analytic data set contained 1 observation for each physician group, with measures of patient panel characteristics from 2010 and 2011 and measures of ACO participation from 2012 through 2014. To estimate the association between a physician group’s ACO participation and the characteristics of their patients, we used multivariable linear regression. Our dependent variable was ACO participation. We used patient panel characteristics as our main independent variables to test for the association of physician group ACO participation with those characteristics. All 4 panel characteristics were included in the same regression. In all regressions, we controlled for the physician group characteristics described above. Regressions were estimated with and without patient weights. Those without weights estimate the likelihood that a physician group participates in an ACO given its patient panel. Those with weights give more weight to larger physician groups and less weight to smaller groups and thus reflect how the differences in physician groups’ participation are associated with individual patients, rather than physician groups. All regressions accounted for clustering of observations within hospital service area, as defined by the Dartmouth Atlas of Health Care, to produce robust SEs.^26^

Each regression was first run for the full sample of physician groups. We then stratified by the year that a physician group began participating in the ACO (2012, 2013, or 2014). Stratified regressions included all physician groups that never participated in an ACO. Statistical analyses were performed using Stata version 14.1 (StataCorp). A 2-sided *P* ≤ .05 was considered to be statistically significant.

## Results

We included a total of 67 891 physician groups caring for 5 394 181 patients. Of these physician groups, 7215 (10.6%) participated in an MSSP ACO by 2014, with the largest group entering the MSSP in its first year, 2012 (Table 1).Table 2 displays the unadjusted characteristics of physician groups’ patient panels and of physician groups stratified by whether the group participated in an MSSP ACO. By mean value across practices, compared with groups not in an ACO, those in an ACO had a greater proportion of black patients (12.1% vs 10.6%), dually enrolled patients (20.8% vs 17.7%), and clinically high-risk patients (34.2% vs 30.2%). The physician groups in an ACO had fewer specialists (23.2% vs 37.7%) and had more physicians who were affiliated with a health system (4.2% vs 3.6%) but fewer who were hospital based (4.5% vs 4.9%). Accountable care organization practices were also larger (658 of 7215 [9.1%] of ACO physician groups had 10 or more physicians in their group, compared with 3236 of 60 676 [5.3%] of non-ACO physician groups).

**Table 1.** Rates of Participation in ACOs Among 67 891 Physician Groups Nationally, 2012-2014

Characteristic	Physician Groups, No. (%)
ACO participation (2012-2014)	7215 (10.6)
Began participating	
2012	3038 (4.5)
2013	2255 (3.3)
2014	1922 (2.8)

Abbreviation: ACO, accountable care organization.

**Table 2.** Baseline Characteristics of Physician Groups Participating in an ACO by 2014 Compared With Physician Groups Not Participating in an ACO^a^

Characteristic	Physician Group	
	In an ACO (n = 7215)	Not in an ACO (n = 60 676)
Characteristics of physician groups' patient panels		
Black	12.1 (19.2)	10.6 (18.1)
Dually enrolled in Medicare and Medicaid	20.8 (23.0)	17.7 (19.3)
Living in high-poverty zip code	10.7 (5.4)	11.1 (5.1)
High risk^b^	34.2 (14.7)	30.2 (16.2)
Characteristics of physician groups		
Specialists in group	23.3 (0.4)	37.7 (0.5)
Physicians with health system affiliation in group	4.2 (0.2)	3.6 (0.2)
Physicians with hospital affiliation in group	4.5 (0.2)	4.9 (0.2)
Physicians in group, No. (%)		
1-2	5218 (72.3)	46 652 (76.9)
3-9	1339 (18.6)	10 789 (17.8)
10-24	348 (4.8)	2136 (3.5)
25-49	146 (2.0)	624 (1.0)
50-99	97 (1.3)	268 (0.4)
≥100	67 (0.9)	207 (0.3)

Abbreviation: ACO, accountable care organization.

^a^ Data are presented as mean (SD) percentage of patients panels’ unless otherwise indicated. Patient characteristics were measured in 2010 to 2011, before ACO participation began.

^b^ Charlson Comorbidity Index of 3 or more (final risk scores range between 0 and 41, with higher values associated with higher mortality risk over 10 years).

In multivariable regression, physician groups with a higher proportion of dually enrolled patients and high-risk patients were more likely to participate in an ACO (Table 3). Physician groups that participated in an ACO had 5.1 percentage points (95% CI, 0.1-10.0 percentage points; *P* = .05; with an average rate in the baseline year of 17.9%) more dually enrolled patients and 4.0 percentage points (95% CI, 1.9-6.1 percentage points; *P* < .001; on a baseline rate of 30.2%) more high–clinical risk patients. At a patient level, patients who were high risk were more likely to be attributed to a group that became part of an ACO, with 4.5 percentage points (95% CI, 0.5-8.5 percentage points; *P* = .03) more high-risk patients being attributed to an ACO. The association between all other measures of patient characteristics and attribution to an ACO were not statistically different from zero.

**Table 3.** Differences in Patient Panel and Patient Characteristics Between ACO-Participating Physician Groups and Nonparticipating Physician Groups^a^

Characteristics of Physician Groups’ Patient Panels	Physician-Group Level (n = 67 891)		Patient Level (n = 5 394 181)	
	Percentage Point Difference in Patient Characteristic With ACO Participation (95% CI)	*P* Value	Percentage Point Difference in Patient Characteristic With ACO Participation (95% CI)	*P* Value
Black	0.7 (−4.38 to 5.7)	.80	−0.3 (−7.4 to 6.9)	.94
Dually enrolled	5.1 (0.1 to 10.0)	.05	4.5 (−2.5 to 11.6)	.21
Living in high-poverty zip code	7.4 (−15.5 to 30.3)	.53	−7.6 (−45.6 to 30.4)	.70
High risk	4.0 (1.9 to 6.1)	<.001	4.5 (0.5 to 8.5)	.03

Abbreviation: ACO, accountable care organization.

^a^ All regressions adjust for the number of physicians in the physician group, the percentage of physicians who were specialists, the percentage of physicians affiliated with a health system, and the percentage of physicians who were hospital based and included hospital service area fixed effects.

Finally, when testing whether these associations differed by the year a physician group became a part of an ACO, we found similar associations across ACO-participation years (Table 4). Groups that began participating in an ACO in 2012 cared for 2 percentage points (95% CI, 0.3-3.7 percentage points; *P* = .02) more high-risk patients compared with non-ACO groups during the same period. At a patient level, this association remained, with 3.5 percentage points (95% CI, 0.8-6.2 percentage points; *P* = .01) more high-risk patients being attributed to ACO-participating physician groups compared with non-ACO groups. Physician groups that began participating in the ACO in 2013 and 2014 were also more likely to care for high-risk patients than non-ACO groups (by 1.5 percentage points in 2013 [95% CI, 0.4-2.5 percentage points; *P* = .006] and 1.0 percentage points in 2014 [95% CI, 0.1-1.9 percentage points; *P* = .03]). However, at a patient level there were no statistically significant differences in the characteristics of patients attributed to ACO-participating groups for any patient characteristic in these later cohorts.

**Table 4.** Differences in Patient Panel and Patient Characteristics Between ACO-Participating Physician Groups and Nonparticipating Physician Groups by Initial Year of Participation^a^

Characteristics	Physician-Group Level		Patient Level	
	Percentage Point Difference in Patient Characteristic With ACO Participation (95% CI)	*P* Value	Percentage Point Difference in Patient Characteristic With ACO Participation (95% CI)	*P* Value
2012 Cohort				
Black	−0.2 (−4.9 to 4.5)	.94	−1.2 (−6.6 to 4.1)	.65
Dually enrolled	4.0 (−1.0 to 8.9)	.12	3.6 (−2.3 to 9.5)	.23
Living in high-poverty zip code	15.1 (−7.1 to 37.3)	.18	11.3 (−14.9 to 37.4)	.40
High risk	2.0 (0.3 to 3.7)	.02	3.5 (0.8 to 6.2)	.01
No.	63 714	NA	4 801 631	NA
2013 Cohort				
Black	0.8 (−2.7 to 4.3)	.65	1.4 (−5.2 to 8.0)	.67
Dually enrolled	1.6 (−0.5 to 3.6)	.13	1.1 (−2.9 to 5.1)	.58
Living in poverty	−7.4 (−19.3 to 4.5)	.22	−18.3 (−45.5 to 8.9)	.19
High risk	1.5 (0.4 to 2.5)	.006	−0.1 (−2.5 to 2.4)	.94
No.	62 931	NA	4 746 511	NA
2014 Cohort				
Black	0.2 (−1.4 to 1.7)	.85	−0.5 (−4.0 to 3.1)	.80
Dually enrolled	0.5 (−0.8 to 1.7)	.48	0.9 (−2.3 to 4.0)	.59
Living in poverty	−0.3 (−8.2 to 7.6)	.94	−4.5 (−30.1 to 21.1)	.73
High risk	1.0 (0.1 to 1.9)	.03	1.7 (−0.6 to 4.0)	.15
No.	62 598	NA	4 694 541	NA

Abbreviations: ACO, accountable care organization; NA, not applicable.

^a^ All regressions adjust for the number of physicians in the physician group, the percentage of physicians who were specialists, the percentage of physicians affiliated with a health system, and the percentage of physicians who were hospital based and included hospital service area fixed effects.

## Discussion

We found little evidence that ACO-participating physician groups are less likely to care for socially vulnerable patients than nonparticipating ACO groups. Physician groups participating in the MSSP ACOs cared for patients who had more comorbidities than nonparticipating physician groups. However, for other patient characteristics, including race, income, and dual eligibility, ACO-participating physician groups closely resembled nonparticipating physician groups. Researchers and policy makers have raised concerns that ACOs may systematically exclude physicians who care for a disproportionate share of socially vulnerable patients.^17,18,19^ Early research on ACOs found that patients attributed to Medicare ACOs tended to have higher incomes and were less likely to be black or enrolled in Medicaid^27^ and that ACOs tended to be located in areas with lower poverty levels.^20^ Other research found that even when controlling for the differences in the geographic location of ACOs, physicians working in zip codes with a higher share of vulnerable patients are less likely to participate in an ACO.^21^ However, that research did not examine the patients attributed to ACO-participating groups, only the characteristics of patients living in the zip codes where the physician groups were located.

In this study, we extend that earlier work by looking at the characteristics of patients who were attributed to each physician group, and we found no evidence to support differences in patient characteristics by ACO participation. When we examined physician group–level characteristics, we found that those groups with higher proportions of dually enrolled patients were more likely to participate in the MSSP ACO program. At the same time, groups with higher proportions of patients who were black or living in high-poverty level zip codes were equally likely to participate. When we evaluated at the patient level, the association for dually enrolled patients was nonsignificant, suggesting that physician groups that care for fewer dually enrolled patients also care for fewer patients in general. Although we found no association at the patient level in social vulnerability and ACO attribution, there was an association between patient illness severity and attribution to an ACO, with patients who had more comorbidities being more likely to be attributed to an ACO-participating physician group. The ACO-participating physicians may be more focused on effectively managing chronic illnesses and better equipped to do so.^28^

There are a number of reasons that practices caring for more chronically ill patients may be more likely to participate in an ACO. First, as noted, ACO practices may be better equipped to manage chronic diseases owing to the population-based emphasis of ACOs, which works to improve care outside the health care setting and also care for patients across health care transitions. Second, because ACO performance is judged against local historical benchmarks for spending, patients with chronic illnesses who tend to consume more health care than average may be an easier target for decreasing health care spending. Improving the efficiency of care for such patients may thus be seen as a viable target to a physician group considering ACO participation, especially if the patient panel of a physician’s practice is sicker than the average population, with room to improve their health care management. Although targeting efficiency gains to patients with chronic conditions is likely good for patients in the short term, the downside is that any efficiency gains from improving the treatment of patients with suboptimally managed chronic illness will likely only produce a 1-time gain in efficiency.

If practices are seeking opportunities to improve by targeting patients among whom efficiency gains will be easier to achieve, this may paradoxically protect vulnerable patients from exclusion from ACOs. That is, given the long history of low quality of care delivered to vulnerable patients, there are opportunities to improve that care and thus improve quality and efficiency of care.

Although our findings are encouraging with respect to the consequences of ACOs on disparities, whether vulnerable patients will equally benefit from being attributed to practices that are participating in the MSSP ACO program remains unknown. We examined baseline characteristics of practices that eventually participated in an ACO, that is, we examined patient characteristics approximately 1 year before ACO participation. After a physician group becomes part of an ACO, the makeup of its patients may change. Preferentially treating healthier or less vulnerable patients would be a strategy to improve performance under an ACO, although a nefarious one, and is an unintended consequence that must be monitored.

Furthermore, even in the absence of choosing healthier or less vulnerable patients, it is possible that physician groups that care for a disproportionate share of vulnerable patients do not see the same gains in quality that have been demonstrated among ACO-attributed patients more generally.^2,3,4,5,6,7,8,9^ Practices that care for a disproportionate share of vulnerable patients are less likely to adopt Medicare’s annual wellness visit, a strategy to target the treatment of high-risk patients that may be helpful to ACOs.^29^ Thus, despite our findings, the broader question of whether ACOs might worsen disparities in care has not been answered.

### Limitations

Our study results should be interpreted in the context of several limitations. First, our measures of social vulnerability are limited to those in the available data. Although race is accurately coded in Medicare claims, these data do not include ethnicity; thus, we could not examine the association between ethnicity and ACO attribution. In addition, our measure of poverty was necessarily measured at the zip code level rather than at the individual level. Second, we used taxpayer identification numbers to identify physician groups. Although this is standard practice, it does not fully represent the range of functional and economic relationships among physicians. Nonetheless, it is how the MSSP program defines and identifies groups of physicians. Our results are from a single ACO program. Although the MSSP is the largest ACO in the country, there has been an expansion of commercial and Medicaid ACOs; in 2016, the MSSP covered fewer than half of all lives attributed to an ACO in the United States. Thus, our results may not generalize beyond the MSSP ACO.

## Conclusions

We provide the first evidence that we are aware of suggesting that physician groups that participated in the MSSP ACO program cared for an equally great number of socially vulnerable patients. Although this is a favorable outcome for ACOs, learning whether this translates into improved quality of care for such patients and thus narrows disparities requires continued monitoring.
